# Commentary: Vitamin C supplementation for prevention and treatment of pneumonia

**DOI:** 10.3389/fmed.2020.595988

**Published:** 2021-01-20

**Authors:** Harri Hemilä, Elizabeth Chalker

**Affiliations:** ^1^Department of Public Health, University of Helsinki, Helsinki, Finland; ^2^School of Public Health, University of Sydney, Sydney, NSW, Australia

**Keywords:** ascorbic acid, meta-analysis, pneumonia, respiratory tract infections, statistics

In the early literature, vitamin C deficiency was associated with pneumonia (1–3). In a 1979 trial, Pitt and Costrini reported just one case of pneumonia in US Marines administered vitamin C, but seven cases in the placebo group, and concluded that “*no claim of a beneficial effect of vitamin C in preventing viral or bacterial pneumonia should be made on the basis of this study, although this certainly should be an area for further investigation*” (4). Two decades later, Hemilä carried out a meta-analysis of three controlled trials assessing the influence of vitamin C on the risk of contracting pneumonia, and concluded that “*the considerably lower pneumonia incidence in the vitamin C groups indicates that further work should be performed to address the question of whether vitamin C affects susceptibility to pneumonia more explicitly*” (5).

Recently, Padhani et al. published a Cochrane review on vitamin C and pneumonia (6). On page 21, they refer to the previous Cochrane review on vitamin C and pneumonia (3) incorrectly claiming that we were the authors. We wrote the Cochrane review on vitamin C and the common cold (7). Nevertheless, we are interested in respiratory infections in general. In this commentary, we describe some concerns we have with the Padhani review (6).

In the section “Criteria for considering studies for this review,” Padhani writes “*Types of studies: We included randomized controlled trials (RCTs) and quasi-RCTs*.” However, there is no indication that two of the included trials (8, 9) were RCTs or quasi-RCTs.

Wahed et al. reported “*the sampling method was systematic sampling and every 1st patient was given the intervention and 2nd patient was treated as control from a prepared register*” [(8), p. 78]. There were six active treatment groups: five micronutrients (*N* = 200), vitamin A (*N* = 40), vitamin C (*N* = 40), vitamin E (*N* = 40), folic acid (*N* = 40), and zinc (*N* = 40). In the analysis, a single control group (*N* = 400) was pooled from each of the control groups of the six active treatments. Based on Wahed's description, patients were allocated to vitamin C (*N* = 40) and control (*N* = 40) groups alternately. However, no data is published about the genuine control group (*N* = 40) for vitamin C. Only the pooled control group of 400 patients is shown (8). It is not clear whether all six treatments were carried out at the same time on the same wards over the 3-year study. Thus, there is no certainty that the 400-patient pooled control group is comparable to the 40-patient vitamin C group. Although the allocation may have been alternate, this is not reflected in the published results. Several further concerns with this trial are described in the Supplementary File. Given the numerous methodological and reporting issues, this trial should have been excluded by Padhani.

Khan et al. allocated 111 patients to the vitamin C and placebo groups, but the report does not describe the method of allocation (9). It should not be assumed that a trial is an RCT or quasi-RCT without evidence. Allocation methods in some trials have resulted in comparisons which are not sound. For example, one trial administered vitamin C to students one winter and compared them with a group of different students monitored during the previous winter (10). In the Khan trial, it is not obvious that the two groups were studied at the same time on the same wards. Given Padhani's inclusion criteria, the Khan trial should also have been excluded.

Padhani further writes “*Types of participants: We included studies involving: 1. healthy adults and children receiving vitamin C supplementation for the prevention of pneumonia*.” However, Coulehan (11) and Bancalari et al. (12) studied vitamin C for the common cold and neither mentioned pneumonia even as a secondary outcome. Furthermore, Padhani extracted the results of both trials incorrectly.

Padhani writes in the Results section [(6), p. 15]: “*Two studies reported incidence of pneumonia (Bancalari 1984; Pitt 1979)… Figure 4*.” According to Padhani's Figure 4, the number of “events” in the Bancalari trial (12) was 21 in each group. This was not the number of infection events, but the number of children who had ≥1 infections. There were 46 respiratory infection events in the placebo group and 38 in the vitamin C group. However, none of these 84 respiratory infections was pneumonia anyway (12) (see the Supplementary File, pp. 23–24).

Padhani also writes: “*Coulehan 1974 did not report on any of our prespecified primary or secondary outcomes*.” Firstly, it is not evident why the trial was included in the analysis if they did not find any data on the prespecified outcomes. Secondly, Coulehan did actually report: “*the school doctor or nurse treated 75 respiratory-illness episodes and 89 other illness episodes… None involved the lower respiratory tract*” [(11), p. 7]. Thus, Coulehan did report “incidence of pneumonia” (a prespecified outcome of Padhani) [(6), p. 8] and the incidence was zero in both groups.

Concerns with the included and excluded trials, and with the risk of bias assessment, statistical calculations, GRADE assessment and the presentation of the summary of findings table are described in our Supplementary File. In addition, Padhani did not include a comprehensive discussion of the previous research on vitamin C and pneumonia, and evidence for the safety of vitamin C. This lack of rigor has also been seen in some other Cochrane reviews (13, 14).

In this commentary we have pointed out several flaws in the recent Cochrane review on vitamin C and pneumonia by Padhani et al. (6). We agree with their conclusion that “*further good-quality studies are required to assess the role of vitamin C supplementation in the prevention and treatment of pneumonia*,” however, that statement lacks novelty, as it has been made several times over previous decades (2–5). Furthermore, some of the previous proposals for further research indicated specific contexts in which the effect of vitamin C should be investigated (2, 3). This discussion was lacking in Padhani's review (6).

## Author Contributions

Both authors contributed to the article and approved the submitted version.

## Conflict of Interest

The authors declare that the research was conducted in the absence of any commercial or financial relationships that could be construed as a potential conflict of interest.
