# Supplementary material for: Commentary: Vitamin C supplementation for prevention and treatment of pneumonia
Source: Front Med (Lausanne). 2021 Jan 20;7:595988. doi: 10.3389/fmed.2020.595988 (PMC7854566; doi:10.3389/fmed.2020.595988)
Supplement: Supplementary file 1 [file Data_Sheet_1.PDF]

General Commentary:
Vitamin C supplementation for prevention and treatment of pneumonia (2020)

Harri Hemilä and Elizabeth Chalker

Supplementary file

2020-7-27

This is a supplementary file to our General Commentary submitted to Frontiers in Medicine

General commentaries have a word limit, which means that only the most significant concerns can be described in the main commentary text. In this Supplementary file we describe in detail many additional concerns in the Cochrane review on vitamin C for pneumonia by Padhani (2020)

All singular page numbers in the text refer to the pages of the Cochrane review by Padhani (2020):

> Padhani ZA, Moazzam Z, Ashraf A, Bilal H, Salam RA, Das JK, Bhutta ZA.
> Vitamin C supplementation for prevention and treatment of pneumonia.
> Cochrane Database of Systematic Reviews 2020, Issue 4. Art. No.: CD013134.
> https://doi.org/10.1002/14651858.CD013134.pub2

When pages of other publications are referred to, the author (year) are mentioned along with the pages.

Contents **Page**

Errors and other weaknesses in the Abstract 2
Errors and other weaknesses in the Summary of Findings (SOF) table 5
Arbitrary GRADE scoring in the SOF table 8
Shortcomings in the search for studies and application of inclusion/exclusion criteria 10
Shortcomings in the assessment of risk of bias 14
Errors and inconsistencies in data extraction 23
Errors and inappropriate methods in statistical calculations 25
Further errors in the description of individual trials 30
Superficial description of the relevant literature 31
Lack of rigor in the Cochrane review on vitamin C for pneumonia 32
References 33

Errors and other weaknesses in the Abstract

In the Abstract (p. 1-2), Padhani writes:

"We also checked references to identify additional studies"

A previous Cochrane review on vitamin C and pneumonia listed 6 included trials and 11 excluded trials (Hemilä 2013a). Although different authors use differing inclusion criteria, the trials listed in the previous version of the review should be listed so that the reader can understand the divergence between the old and new versions of the review. In the characteristics of excluded studies list, Padhani lists several papers that are obviously irrelevant for the review and should not be listed according to the Cochrane guidelines (pp. 10-13 of this Supplement).

"Selection criteria: We included ... trials ... assessing the role of vitamin C supplementation in the prevention and treatment of pneumonia".

Coulehan (1974) and Bancalari (1984) were included, though they did not assess the role of vitamin C on pneumonia (pp. 21,23-24 of this Supplement). Only 30% of the patients in the Hunt (1984) trial were diagnosed by the authors as having pneumonia, yet the trial was included anyway (p. 12-13 of this Supplement).

"We included seven studies in the review and identified two ongoing studies. The seven included studies involved a total of 2774 participants; five studies were RCTs and two were quasi-RCTs."

One of the five studies that Padhani claims to be a RCT used alternative allocation (Coulehan 1974) and was thus not a RCT, but a quasi-RCT (p. 14 of this Supplement). The two quasi-RCT trials mentioned in the above sentence either did not report the method of allocation (Khan 2014) (p. 15 of this Supplement), or used alternative allocation but did not publish the results of the parallel control group and had many other severe flaws (Wahed 2008)(p. 11-12,15,20 of this Supplement).

Furthermore, two of the seven trials mentioned in the above sentence did not assess the role of vitamin C for pneumonia (Coulehan 1974, Bancalari 1984) (p. 21,23-24 of this Supplement).

Thus, only 3 of the 7 included trials were RCTs or quasi-RCTs assessing the effect of vitamin C on pneumonia.

*"For pneumonia prevention, the included studies provided supplementation in doses of 500 mg daily for 14 weeks, 2 g daily for 8 weeks, and 2 g daily for 12 weeks" (*Abstract, p. 1).

The 500 mg daily vitamin C for 14 weeks refers to the Coulehan (1974) trial; each tablet contained 500 mg of vitamin C, however, the actual doses used by Coulehan were 1 g daily for younger children and 2 g per day for older children (p. 30 of this Supplement).

"Three studies assessed the effect of vitamin C supplementation for pneumonia prevention"

The three studies to which Padhani refers to here are the Coulehan (1974), the Bancalari (1984), and the Pitt (1979) trials. However, the Coulehan and the Bancalari trials did not assess the effect of vitamin C supplementation for pneumonia prevention. Instead, they both assessed the effect of vitamin C supplementation for the common cold. Neither of the two studies reported any cases of pneumonia (pp. 21,23-24 of this Supplement).

"the effect of vitamin C supplementation on pneumonia incidence (risk ratio (RR) 0.46, 95% confidence interval (CI) 0.06 to 3.61; 2 studies, 736 participants"

This RR estimate is based on pooling the Bancalari (1984) and Pitt (1979) trials. However, the clinical context of the two trials was so different that it is not appropriate to pool them (p. 25 of this Supplement). Furthermore, the events in the Bancalari trial were not pneumonia (pp. 23-24 of this Supplement). In addition, the confidence interval was calculated with an inappropriate method (p. 26-28 of this Supplement). Thus the RR estimate that Padhani publishes in the Abstract is meaningless.

"the effect of vitamin C supplementation on ... adverse events (urticaria) (RR 3.11, 95% CI 0.13 to 76.03; 1 study, 674 participants"

This statement is based on the Pitt (1979) trial in which just one case of urticaria occurred in the vitamin C group and 0 cases in the placebo effect. On the basis of such data, it is not reasonable to argue that vitamin C increases the risk of urticaria by 3.11 fold. In addition, the confidence interval was calculated with an inappropriate method (pp. 26-28 of this Supplement).

"Five studies assessed the effect of vitamin C supplementation as an adjunct to pneumonia treatment"

There is no evidence that 2 of the above mentioned 5 studies (Wahed 2008 and Khan 2014) were RCTs or quasi-RCTs and they should not have been included (p. 15 of this Supplement). One of the 5 studies did not report anything about pneumonia (Bancalari 1984) (pp. 23-24 of this Supplement).

"One study reported a decrease in the duration of illness in the vitamin C supplementation group (3.4 days ± 2.54) compared to the control group (4.5 days ± 2.35),"

This sentence refers to the Bancalari (1984) trial. However, the SD estimates (ie 2.54 and 2.35 days) were incorrectly calculated by Padhani (pp. 25-26 of this Supplement). Furthermore, none of the recorded illnesses were pneumonia (pp. 23-24 of this Supplement).

"one study reported a decrease in number of days required for improvement in oxygen saturation (1.03 days ± 0.16 versus 1.14 days ± 1.0) and respiratory rate (3.61 days ± 1.50 versus 4.04 days ± 1.62) in the vitamin C supplementation group compared to the control group"

These are not primary or secondary outcomes defined in the Methods section.

“the effect of vitamin C supplementation on mortality due to pneumonia (RR 0.21, 95% CI 0.03 to 1.66”

This is based on a small Hunt (1994) trial that reported just six deaths and the confidence interval was calculated with an inappropriate method (pp. 26-28 of this Supplement). In addition, just 30% of the patients in the Hunt trial had pneumonia, and Hunt (1994) did not describe how the six deaths were distributed between the pneumonia and non-pneumonia patients (p. 13 of this Supplement).

“One study reported that the mean duration of hospital stay was 6.75 days amongst children in the vitamin C supplementation group and 7.75 days in the control group”

This refers to the Wahed (2008) trial which had such severe shortcomings that it should not be included (pp. 11-12,15,20 of this Supplement; see also the main text).

Errors and other weaknesses in the Summary of Findings (SOF) table

<u>The first section (p. 4-5): *"Vitamin C compared to placebo for prevention of pneumonia"*</u>

The participants of the included trials are described as follows:
*"Patient or population: school-aged children (10 to 15 years) and marine corps recruits
Settings: Chile, USA, UK"*

In fact, none of the trials on vitamin C for prevention of pneumonia was carried out in the UK.

In the SOF table (p. 4), Padhani describes that the events counted for "prevention of pneumonia" were *"Defined as patient admitted to hospital with a diagnosis of pneumonia"*. However, Bancalari et al. did not report any cases of pneumonia in their trial, and thus, there were no reported admissions to hospital with a diagnosis of pneumonia (pp. 23-24 or this Supplementary file).

The section of the SOF table shows *"illustrative comparative risks"* and gives the *"assumed risk"* of incidence of pneumonia in the placebo group as "75 per 1000" and in the vitamin C group as "61 per 1000". There is no description of where the figure 75 comes from. Neither is there a description for the unit of the 1000; it may be persons or person years.

In the general Western adult population, the incidence of pneumonia is 1-3 cases per 1000 person years (Merchant 2004, Hemilä 2006), so that the risk assumed by Padhani is very high if it indicates 75 cases of pneumonia per 1000 person years in the ordinary population.

In the Pitt (1979) trial which is the "marine corps recruits" trial, there were 7 cases of pneumonia in 343 participants given placebo during the 8 week follow-up. This would lead to an assumed average incidence of 0.0204 per participant per 8 weeks, and 0.132 per participant per year, and 132 per 1000 person-years. Thus, Padhani's high level of assumed risk may be justified if the focus is only marine or military recruits. Nevertheless, it is not reasonable to base an "illustrative comparative risk" on a study in very special conditions observing just 7 cases.

Padhani pooled *"school-aged children (10 to 15 years) and marine corps recruits"* together. The study in school-aged children was the Bancalari (1984) trial, which did not observe any cases of pneumonia in either the vitamin C or placebo group (pp. 23-24 of this Supplement).

For the *"Patient or population: school-aged children (10 to 15 years) and marine corps recruits"*, the SOF table gives the estimate of effect RR = 0.46 (95% CI 0.06 to 3.61).

This effect estimate is based on pooling the Pitt (1979) and Bancalari (1984) trials in Analysis 1.1 (Figure 4). However, the trials are clinically so different that it is inappropriate to pool them (p. 25 of this Supplementary file). Furthermore, for the incidence of pneumonia, Padhani extracted incorrect data from the Bancalari (1984) report (pp. 23-24 of this Supplement; see also the main text).

Finally, the RR = 0.46 is inconsistent with the assumed and corresponding risks by Padhani which give RR = 0.81 (= 61/75).

In the SOF table (p. 5), Padhani writes "*The basis for the assumed risk (e.g. the median control group risk across studies) is provided in footnotes. The corresponding risk (and its 95% confidence interval)*

is based on the assumed risk in the comparison group and the relative effect of the intervention (and its 95% CI)."
However, no basis for "assumed risk" is given in the footnotes and the corresponding risk is not calculated from the assumed risk and the relative effect of vitamin C.

The second section (pp. 5-6): *"Vitamin C compared to placebo for treatment of pneumonia"*

The participants of the included trials are described as follows:
"Patient or population: children under 5 years of age and adults
Settings: UK and Pakistan"

The Bancalari (1984) trial results are shown in this section, however, that trial was not carried out in the UK or Pakistan. It was carried out in Chile.

In the Cochrane review, Padhani et al. do not define what they mean by the term "treatment". Usually "treatment of pneumonia" indicates that first a person starts to suffer from symptoms suggesting pneumonia, and after examinations leading to diagnosis, treatment is initiated. However, the Bancalari (1984) trial was not such a "treatment trial".

Bancalari administered healthy children 2 grams per day of vitamin C and recorded the number and duration of respiratory infections that occurred after the start of the 12-week daily vitamin C administration. Although it is possible to test the effects of vitamin C on the duration and severity of respiratory infections in such a setting, it is not a "treatment trial" in the usual sense.

The first row of the second section of the SOF table shows the effect of vitamin C on the duration of illness in the Bancalari (1984) trial. The SOF table states that the number of participants was 130, whereas Bancalari (1984) reported *"The 62 students were divided into two random groups: the vitamin C group (N=32) and the placebo group (N=30)"*.

The results of the Bancalari (1984) trial are shown in the SOF table as follows:
"Placebo: Mean duration of illness was 4.5 days (± 2.35)."
"Vitamin C: Mean duration of illness was 3.4 days (± 2.54)."

First, these mean illness duration values are not for pneumonia (pp. 23-24 of this Supplement). Second, Padhani calculated the SD values incorrectly (pp. 25-26 of this Supplement).

The second and third rows of the second section of the SOF table shows the effect of vitamin C on the number of days for improved oxygen saturation from the Khan (2014) trial. However, there is no evidence that the Khan trial was RCT or quasi-RCT and therefore it should not be included when using the inclusion criteria of Padhani (p. 15 of this Supplement). Furthermore, "number of days for improved oxygen saturation" was not pre-specified as a primary or secondary outcome by Padhani.

The fifth row of the second section of the SOF table shows the effect of vitamin C on mortality of pneumonia and acute bronchitis patients of the Hunt (1994) trial. That row shows *"illustrative comparative risks"* and gives the *"assumed risk"* in the placebo group as "72 per 1000" and in the vitamin C group as "36 per 1000". However, there is no description of where the figures 72 and 36 come from. Nor is there a description of the units for the 1000.

In the Hunt (1994) trial, there were 5 deaths due to lower respiratory infections in 29 placebo patients during the 4 week follow-up after hospital admission. This would lead to an assumed average mortality rate of 0.172 per patient per hospital treated (30% pneumonia and 70% acute bronchitis), and 172 per 1000 patients. Here again, there is no transparency regarding where the 72 shown in Padhani's SOF table comes from. It is not sound to base an "illustrative comparative risk" on observing just 5 deaths in patients under special conditions. However, if there is some other rationale for the illustrative comparative risk, it should be described to the readers.

For "Mortality due to pneumonia", the SOF table gives an estimate of effect of RR = 0.21 (95% CI 0.03 to 1.66). This confidence interval was calculated using an inappropriate method (pp. 26-28 of this Supplement). In addition, it is highly questionable whether any confidence interval that is based on 1 and 5 deaths is meaningful for the SOF table context. Finally, the RR = 0.21 is inconsistent with the assumed and corresponding risks by Padhani which give RR = 0.5 (= 36/72).

In SOF (p. 6), Padhani writes "*The basis for the assumed risk (e.g. the median control group risk across studies) is provided in footnotes. The corresponding risk (and its 95% confidence interval) is based on the assumed risk in the comparison group and the relative effect of the intervention (and its 95% CI).*"
However, no basis for "assumed risk" is given in the footnotes and the corresponding risk is not calculated from the assumed risk and the relative effect of vitamin C.

Arbitrary GRADE scoring in the SOF table

In the SOF table, Padhani rates the evidence for the effect of vitamin C on "Incidence of pneumonia" as "very low". Padhani justifies this scoring by the following arguments *a)* to *d)*:

"a) Downgraded by one level due to study limitations (unclear sequence generation and allocation concealment and high risk of attrition bias)."

However, in the risk of bias assessment, Padhani (p. 28) is fully satisfied with the sequence generation in the Pitt (1979) trial:
Pitt (1979): *"Random sequence generation (selection bias) Low risk*
Quote: "Recruits were randomly assigned to either of the group from a list of number in random pairs." (p. 908)
Comment: adequately done"

Pitt (1979) reported that the trial was double-blind and logically that means that allocation was concealed (pp. 16-17 of this Supplement).

Finally, the number of drop-outs were very similar in both groups of the Pitt trial. Many of the exclusions were a result of administrative decisions to relocate participants. There is no substantial baseline difference between the trial arms (p. 20 of this Supplement).

Therefore this downgrading is not justified.

"b) Downgraded by one level due to high heterogeneity (I^2 = 75%)."

Padhani pooled the Bancalari (1984) trial in schoolchildren aged 10 to 12 years with the Pitt (1979) trial with US Marine recruits. The participants and contexts are so different that it is scientifically unsound to pool them (p. 25 of this Supplement). In addition, the data that Padhani extracted from the Bancalari (1984) trial was not about pneumonia (pp. 23-24 of this Supplement). Thus, heterogeneity between the Pitt (1979) and the Bancalari (1984) trials is caused by pooling incompatible trials and extracting incorrect data from the Bancalari trial. In this case, the test of heterogeneity does not measure heterogeneity in the effect of vitamin C on pneumonia.

Therefore this downgrading is not justified.

"c) Downgraded by one level due to small sample size."
"d) Downgraded by one level due to imprecision (wide CI)."

"Small sample size" and "imprecision" are two different names for the same issue. It is not appropriate to use the same argument twice just by using a different name for the argument.

Therefore one of these downgradings is not justified.

Furthermore, GRADE also allows the scores to be upgraded. For the Pitt (1979) trial, the OR was incorrectly calculated by Padhani. Calculated correctly, OR = 0.23 (95% CI: 0.06 to 0.92) (p. 27 of this Supplement).

The Cochrane Handbook (2019, sect 14.2.1) instructs that: "*In GRADE, a body of evidence from randomized trials begins with a high-certainty rating*".
GRADE allows upgrading if there are large effects (sect 14.2.3):

> *14.2.3 Domains that may lead to increasing the certainty level of a body of evidence*
>
> *Although NRSI and downgraded randomized trials will generally yield a low rating for certainty of evidence, there will be unusual circumstances in which review authors could 'upgrade' such evidence to moderate or even high certainty (Table 14.3.a). ...*
>
> *Large effects On rare occasions when methodologically well-done observational studies yield large, consistent and precise estimates of the magnitude of an intervention effect, one may be particularly confident in the results. A large estimated effect (e.g. RR >2 or RR <0.5) in the absence of plausible confounders, or a very large effect (e.g. RR >5 or RR <0.2) in studies with no major threats to validity, might qualify for this.*

Thus, if Padhani had focused just on the Pitt (1979) trial, by excluding the Bancalari (1984) trial as it does not contribute any information about pneumonia, the GRADE assessment could have been "high quality" based on downgrading by 1 point because of the imprecision and upgrading by 1 point because the observed effect was large (RR < 0.5).

Obviously, the GRADE scoring does not change Pitt's (1979) conclusion that *"no claim of a beneficial effect of vitamin C in preventing viral or bacterial pneumonia should be made on the basis of this study, although this certainly should be an area for further investigation"*. Nevertheless, "high quality evidence" gives much more weight to the claim for the need for further investigation than Padhani's "very low quality evidence".

Shortcomings in the search and inclusion/exclusion of studies

Padhani writes "*Searching other resources: We also checked reference lists of primary studies and reviews for additional references*" (p. 9) and "*we attempted to be as inclusive as possible in our searches"* (p. 17).

The previous Cochrane review on vitamin C and pneumonia by Hemilä (2013a) included 6 trials and listed 11 trials on the exclusion list, so that the total number of listed trials was 17. Ideally authors of a new Cochrane review should look at the previous version of the review on the same topic for additional references, but that was not done by Padhani. Ten of the 17 trials in the 2013 version are not considered by Padhani. Evidently, there are differences in inclusion criteria and there is room for subjective interpretation and so identical lists of included trials should not be expected; however, the trials included in an earlier version should be accounted for as included or excluded with an explanation so that the reader can understand the reasons for the differences between the reviews.

This lack of considering the references in the previous review on the same topic is inconsistent with the The Methodological Expectations of Cochrane Intervention Reviews (MECIR), which states that it is mandatory to *"Check reference lists in included studies and any relevant systematic reviews identified"* with rationale and elaboration that "*Searches for studies should be as extensive as possible in order to reduce the risk of publication bias and to identify as much relevant evidence as possible*" (MECIR 2020 item C30).

Furthermore, Padhani writes:
"We used standard methodological procedures expected by Cochrane" (Abstract, p. 1)
"We rigorously followed Cochrane methods" (Discussion, p. 17)

That did not take place in the construction of the list of "characteristics of excluded studies" (p. 32)

The Cochrane Handbook (2019) instructs that the excluded trial list should focus on trials that are potentially relevant (section 4.6.5). Yellow is added to emphasize the main points.

"A Cochrane Review includes a list of excluded studies called 'Characteristics of excluded studies', detailing the specific reason for exclusion for any studies that a reader might plausibly expect to see among the included studies. This covers all studies that may, on the surface, appear to meet the eligibility criteria but which, on further inspection, do not. It also covers those that do not meet all of the criteria but are well known and likely to be thought relevant by some readers. By listing such studies as excluded and giving the primary reason for exclusion, the review authors can show that consideration has been given to these studies. The list of excluded studies should be as brief as possible. It should not list all of the reports that were identified by an extensive search. It should not list studies that obviously do not fulfil the eligibility criteria for the review, such as 'Types of studies', 'Types of participants', and 'Types of interventions'. In particular, it should not list studies that are obviously not randomized if the review includes only randomized trials."

In the "characteristics of excluded studies" (p. 32), Padhani lists 20 publications. Seven of them are reviews, three are correspondence, one is a cohort study, and one had no comparison group. Thus, at least 12 of the 20 publications are unambiguously inconsistent with the Cochrane instructions above. They obviously do not fulfil the eligibility criteria for the review.

Furthermore, the Glazebrook (1942) trial was excluded on the basis of the following statement: *“Glazebrook 1942: Wrong study design - the study design was not appropriate since the study recruited new participants in the middle of the study”*.

However, many or most controlled trials recruit participants over a time period. There is no meaningful difference between recruiting new participants over a time period and recruiting in the middle of a trial. In their Methods section, Padhani et al. do not state that trials that recruited over a time period or at two separate time points are excluded. The reasons for exclusion should be consistent with the inclusion and exclusion criteria described in the Methods section.

The Mahalanabis (2006b) trial was excluded on the basis of the following statement: *“Mahalanabis 2006b: Wrong study design - letter to the editor”*.

In their Methods section, Padhani writes *“We included studies reported as full text, abstract only, and unpublished data.”* As a publication type, a letter to the editor is no worse than an abstract only or unpublished data. In some cases letters to the editor are detailed and contain relevant descriptions of trial methods. Therefore, this exclusion statement is inconsistent with the published Methods. The Mahalanabis 2006b trial can be excluded, but it should be excluded for reasons stated in the Methods section.

There are also problems in the inclusion of trials. Our main commentary describes some of the problems in the Wahed (2008) trial, but there are further serious flaws. In their Cochrane review, Hemilä (2013a, pp. 33-34) wrote:

“*The description of the methods of this 7-arm [Wahed 2008] trial is minimal. The dose of vitamin C is not described. It is not clear whether a placebo was used. The authors state that ”initially data was collected from 1150 children and after exclusions only 800 children were selected for analysis.“ However, the original number of children in each of the 7 groups is not reported. When the reasons for exclusion seem to be random (complications of pneumonia etc.) it does not seem possible that random dropping out would lead to 5 groups which each had exactly 40 children and a ~~placebo~~ [control] group which had exactly 400 children. The duration of hospital stay because of pneumonia in the control group (n = 400) was 7.75 d and in the vitamin C group (n = 40) was 7.00 d. However, the SD is not given for the estimates. Due to these and many further problems we excluded the trial*.”

In the Hemilä Cochrane review (2013a, p. 78), the Wahed (2008) trial was excluded for the reasons described above. It would seem appropriate for the authors of a new Cochrane review on the same topic to describe why they disagree with the exclusion of the Wahed (2008) trial in the earlier version of the review. Padhani et al. do not address this at all in their review.

Furthermore, Wahed (2008, p 78) did not follow the intention to treat (ITT) principle. They wrote:

”*The children who left the hospital on ‘risk bond’ or ‘absconded’ from the ward or ‘expired’ during treatment were excluded from the analysis. Also those who developed a complication of pneumonia, were suffering from other severe systemic diseases with pneumonia, required antibiotics other than*

ampicillin and gentamysin and whose parents did not give consent for drawing blood were also excluded from the study."

The Cochrane Handbook (sect 8.2.2) describes [**bold** in the original]:

"*The effect of **assignment** to intervention should be estimated by an **intention-to-treat (ITT) analysis** that includes all randomized participants (Fergusson et al 2002). The principles of ITT analyses are (Piantadosi 2005, Menerit 2012):*
1. analyse participants in the intervention groups to which they were randomized, regardless of the interventions they actually received; and
2. include all randomized participants in the analysis, which requires measuring all participants' outcomes."

Excluding patients on the basis that they had complications or expired, or required other antibiotics, is unambiguously inconsistent with the ITT principle. If vitamin C has an effect on pneumonia patients, then excluding patients by their clinical progress can substantially bias the comparison.

Despite its numerous flaws, the Wahed trial is given great importance in Padhani's review. Padhani's Abstract describes Wahed's results individually as follows: "*One study reported that the mean duration of hospital stay was 6.75 days amongst children in the vitamin C supplementation group and 7.75 days in the control group*".

In the Methods section, Padhani (p. 8) describes in the inclusion criteria:
"We included randomised controlled trials (RCTs) and quasi-RCTs evaluating the following:
2. role of vitamin C supplementation as an adjunct to the treatment of pneumonia."
and
"We included studies involving: 2. adults and children with confirmed pneumonia (as defined by study authors) receiving vitamin C supplementation as an adjunct to the treatment of pneumonia."

In the Methods section, Padhani (p. 8) describes outcomes as follows:
"Primary outcomes: Pneumonia treatment: 3. Mortality due to pneumonia.

Padhani (p. 24) writes about the Hunt (1994) trial:
"Type of study: study on pneumonia treatment"

However, only 30% of the respiratory infections in the Hunt (1994) trial were pneumonia.
Hunt (1994, pp. 871-872) wrote of their participants:
"The patients enrolled into this second phase study were suffering from acute bronchitis (often acute exacerbation of chronic bronchitis) or bronchopneumonia."

More detail of the Hunt participants is shown in their Table VIII (Hunt 1994, p. 217):

Vitamin C group:
Acute bronchitis 19 patients
Bronchopneumonia 9 patients

Placebo group:
Acute bronchitis 21 patients
Bronchopneumonia 8 patients

Totals based on the above figures:
Acute bronchitis 40 patients (70%)
Bronchopneumonia 17 patients (30%)

Thus, only 30% (17/57) of the Hunt (1994) patients suffered from pneumonia. Hunt (1994) does not describe the distinction between acute bronchitis and pneumonia.

It may be appropriate to include the Hunt (1994) trial in a systematic review on vitamin C and lower respiratory tract infections. However, Padhani states in the Methods *"we included studies involving: 2. adults and children with confirmed pneumonia (as defined by study authors)"*. With such inclusion criterion it seems inappropriate to include the Hunt trial given that 70% of the respiratory infections were not pneumonia as defined by study authors. Padhani does not mention anywhere in the Cochrane review that 70% of the respiratory infections in the Hunt (1994) trial were "acute bronchitis (often acute exacerbation of chronic bronchitis)".

Padhani (Analysis 2.1, p. 36) analyzed mortality in the Hunt (1994) trial. There were 6 deaths among the 57 participants. However, Hunt does not show the distribution of the 6 deaths by the diagnosis of the respiratory infection. Thus, it is even possible that all the deaths occurred in the patients with acute bronchitis.

Referring to the Hunt (1994) trial, Padhani writes in the Abstract *"the effect of vitamin C supplementation on mortality due to pneumonia (RR 0.21, 95% CI 0.03 to 1.66; 1 study, 57 participants"*. This misleads readers as it gives an impression that the RR estimate and the 57 participants were unambiguous pneumonia cases.

Thus, the Hunt (1994) trial does not provide appropriate data for Padhani's comparison on (p. 8): *"Primary outcomes: Pneumonia treatment: 3. Mortality due to pneumonia.*

Shortcomings in the assessment of risk of bias

We agree with Padhani (p. 17) that "*the process of assessing risk of bias, for example, is not an exact science and includes many personal judgements.*" Nevertheless, the assessment of risk of bias should be consistent with the available facts.

Random sequence generation (selection bias)

Bancalari (1984) and Hunt (1994) trials

In the risk of bias summary (Figure 2, p. 14), Padhani assessed "random sequence generation" as "unclear risk" for both the Bancalari (1984) and Hunt (1994) trials.

Bancalari (1984, p. 871) wrote in the Materials and Methods section:
"The 62 students were divided into two random groups: the vitamin C group (n=32) and the placebo group (n=30). The vitamin C tablets (2 g per day) and the placebo tablets were identical in color, taste, size and consistency, and were marked with codes understood only by staff members in the Department of Applied Biochemistry of the University of Concepción. Like the children, those who collected the data did not know who was taking vitamin C and who was taking the placebo (i.e., it was a double-blind study)."

Although Bancalari (1984) does not report the technical method for generating the random code, the above description does not indicate any concern regarding selection bias. Furthermore, similarity of baseline variables can support the success of allocation. Sex and age were reported and balanced in the treatment groups. There is no evidence to classify selection bias as an "unclear risk".

Hunt (1994, p. 213) wrote in the Materials and Methods section:
"After the initial clinical assessment and blood sampling the patients commenced placebo or vitamin C therapy to which they were allocated on a randomised 'double-blind' basis."

Although Hunt (1994) also does not report the technical method for generating the random code, the above description does not indicate any concern regarding selection bias. Furthermore, similarity of baseline variables support the success of allocation. In the Hunt trial, sex and age (Table II), vitamin C levels in plasma, polymorphonuclear cells and mononuclear cells (Table III), and total respiratory clinical score (Table V) were reported and balanced in the treatment groups. Again, there is no evidence to classify selection bias as an "unclear risk".

Coulehan (1974)

Padhani (p. 12) writes about the Coulehan (1974) trial:

"Included studies: We included seven studies ... Five studies were RCTs (... Coulehan 1974 ...)"

Coulehan (1974, p. 7) wrote:
"All children were assigned alternately, from an alphabetical listing by classroom, to one of two study groups"
Thus the trial was not a RCT, but a quasi-RCT.

Oddly enough, on page 15, Padhani writes *"Coulehan 1974 ... used alternative assignment techniques,"*

Khan (2014)

In the characteristics of included studies, Padhani (p. 26) writes about the Khan (2014) trial:
"Random sequence generation (selection bias): High risk
Randomisation not done".

In the Results section, Padhani (p. 15) writes "*Khan 2014 did not randomise participants*".

In the Patients and Methods section, Khan (2008, p. 56) does not give any indication about the method of allocation. Some trials have allocated participants such that the patient groups were not comparable. For example, in their study on respiratory infections, Gorton (1999) administered vitamin C to students one winter and compared them with a different group of students monitored during the previous winter. There is great variation in the distribution of viruses between different winters and variation in the susceptibility of different people. Therefore, such a study design is not meaningful. Because such flawed study designs are used and published, it should not be assumed that a trial is a RCT or quasi-RCT when there is no description of the allocation methods. In the Khan trial, it is not clear that the two groups were studied in parallel with participants selected from the same pool.

Given that Padhani's inclusion criteria includes the requirement (p. 8) that trials should be "*randomised controlled trials (RCTs) and quasi-RCTs*", it is not appropriate to include a trial for which the method of allocation is unclear. In the characteristics of included studies, Padhani does not describe any evidence from the Khan (2014) trial report to suggest that it was even a quasi-RCT.

Wahed (2008)

In the Results section, Padhani (p. 15) writes about the Wahed (2008) trial:
"Wahed 2008 used alternative assignment techniques"

In the characteristics of included studies, Padhani (p. 30) writes about the Wahed (2008) trial:
"Random sequence generation (selection bias): High risk
Quote: "The sampling method was systematic sampling and every 1st patient was given the intervention and 2nd patient was treated as control from a prepared register."
Comment: not adequately done".

This is misleading. Wahed et al. (2008) described that they used alternative allocation, but reported the results for 6 active treatment arms, but only for 1 control group pooled from the control groups of all the 6 active treatment groups. There are no data about the vitamin C control group and therefore the alternative allocation did not materialize as published parallel control group data for vitamin C treatment (p. 11-12 of this Supplement; see also the main text). The use of alternative allocation is irrelevant when the results are not published by the allocated groups.

Yaqub (2015)

In the risk of bias graph (Figure 3, p. 14), Padhani marks "low risk" for the risk of bias item "random sequence generation" for the Yaqub (2015) trial.

In the characteristics of included studies, Padhani (p. 31) writes about the Yaqub (2015) trial:

"Random sequence generation (selection bias): Low risk.
Quote: "All children with pneumonia were randomised based on lottery method into two groups. Lottery method was used to randomise participants." (p. 210)
Comment: adequately done"

In the Results section, Padhani (p. 15) writes about the Yaqub (2015) trial:
"We assessed two studies to be at low risk of bias for sequence generation (Pitt 1979; Yaqub 2015) ... Yaqub 2015 employed a lottery method for randomisation."

However, Yaqub et al. (2015, p. 209) also write in their Methods section *"... using Non-probability consecutive sampling technique."* This is ignored by Padhani in their description of the Yaqub (2015) trial.

It is not evident what kind of allocation method Yaqub actually used. "Non-probability consecutive" might indicate alternative allocation.

Instead of Padhani's assessment of "low risk" for the risk of bias item "random sequence generation" for the Yaqub (2015) trial, the option "unclear risk" would seem much more appropriate.

Allocation concealment

Bancalari (1984), Coulehan (1974), Hunt (1994), and Pitt (1979)

In the risk of bias summary (Figure 2, p. 14), Padhani marks "unclear risk" for the risk of bias item "allocation concealment" for the Bancalari (1984), Coulehan (1974), Hunt (1994), and Pitt (1979) trials.

In the Results section, Padhani writes (p. 15):
"Allocation concealment: … We assessed five studies to be at unclear risk of bias due to insufficient information regarding allocation concealment (Bancalari 1984; Coulehan 1974; Hunt 1994; Pitt 1979; Yaqub 2015)."

"Allocation concealment" means that participants and researchers are not aware of the group to which participants are allocated at the stage of randomization (or quasi-randomization). For example, in surgery it is essentially always impossible to hide the treatment stage from patients and physicians, but nevertheless, the allocation stage can be blinded so that patients and researchers do not know to which group a certain patient is being allocated.

Double-blinding cannot be done for all treatments, but when it is done, it logically always leads to allocation concealment. Otherwise there could not be blinding at the later stages of the trial.
This reasoning follows basic logic called "syllogism". The standard example of syllogism is as follows: All men are mortal, Socrates is a man, therefore Socrates is mortal.

The argument on double-blinding and allocation concealment is simple logic as follows:

- Double-blinding means that over all time points of a controlled trial, from the very beginning to the very end, all participants and researchers are unaware of the treatment a particular patient is receiving.

- Allocation is one time point within a controlled trial (at the very beginning). Therefore, the existence of double-blinding implies that participants and researchers are unaware of the treatment at the time point of allocation. Thus, double-blinding logically implies allocation concealment.

However, the converse is not necessarily true: mortality of Socrates does not logically imply that all men are mortal. Allocation concealment does not imply double blinding.

Thus, Padhani's question marks in the allocation concealment item are not logical for the double-blind trials. Double-blinding means that there had to be allocation concealment and thus there should be a green plus in the risk of bias item "allocation concealment" for the Bancalari (1984), Coulehan (1974), Hunt (1994), and Pitt (1979) trials in the risk of bias summary (Figure 2, p. 14).

Blinding of participants and personnel (performance bias)

In the risk of bias summary (Figure 2, p. 14), Padhani marks "blinding of participants and personnel" for the Khan (2014) trial as "low risk".

In the characteristics of included studies, Padhani (p. 26) writes about the Khan (2014) trial:
"Blinding of participants and personnel (performance bias): Low risk.
Quote: "In other group children received placebo drops (consisted of sodium citrate along with coloring agent mixed in water), which matched exactly with vitamin C drops in color and taste" (p. 56)".

However, that information is only about the participants. Khan (2014) does not provide any information about the blinding of personnel. Therefore, it would be more appropriate to mark the assessment as "unclear risk".

Blinding of outcome assessment (detection bias)

In the risk of bias summary (Figure 2, p. 14), Padhani marks "blinding of outcome assessment" for the Hunt (1994) trial as "unclear risk":

In the characteristics of included studies, Padhani (p. 25) writes about the Hunt (1994) trial:
"Blinding of outcome assessment (detection bias) All outcomes: Unclear risk
Insufficient information to permit judgement"

In Analysis 2.1 (p. 36) Padhani shows the findings of the Hunt (1994) trial on mortality in elderly hospital patients. Lack of blinding of outcome assessment can cause bias in subjective outcomes, such as when measuring pain or when viewing X-ray images. However, death is an outcome that leaves no room for personal interpretation. It is not reasonable to assume that some deaths in the Hunt trial were undetected because researchers might have known to which group the dead body belonged.

Furthermore, Hunt et al. (1994) described their trial:*"After the initial clinical assessment and blood sampling the patients commenced placebo or vitamin C therapy to which they were allocated on a randomised "double-blind" basis."*
This indicates that outcome assessment was also blinded.

Padhani et al. do not give an explanation as to why they believe that the Hunt trial was not "double-blind" even though they reported it to be so, and how the possible break in blinding of outcome assessment might have biased the counting of bodies.

Classifying "blinding of outcome [mortality] assessment" as "unclear risk" seems inappropriate in the Hunt (1994) trial.

<u>Incomplete outcome data (attrition bias)</u>

Coulehan (1974)

Padhani (p. 15) writes about the Coulehan (1974) trial:
"We judged five studies to be at low risk for attrition bias as there were no reported losses to follow-up ... Coulehan 1974 ..."

Coulehan (1974, p. 6-7) wrote:
"There were 666 children at the outset, ranging in age from six through 15 years... Six hundred and forty-one of the 666 children (96 per cent) completed the entire 14-week study period... 25 subjects (13 C and 12 P) were eliminated from the study because they dropped out of school during its course."

A 3.8% (25/666) drop-out rate is not high, but it is incorrect to describe it to readers as "no reported losses to follow-up".

Hunt (1994)

In the risk of bias summary (Figure 2, p. 14), Padhani marks "incomplete outcome data" for the Hunt (1994) trial as "low risk".

In the characteristics of included studies, Padhani (p. 25) writes about the Hunt (1994) trial:
"Incomplete outcome data (attrition bias): All outcomes: Low risk.
No loss to follow-up"

However, Hunt (1994, p. 214) wrote: *"Full clinical information was obtained on 57 patients at 0, 2 and 4 weeks. Four patients were excluded because of incomplete information."*

Although the rate of 6.6% (4/61) exclusions is not high, it is incorrect to describe it as "no loss to follow-up".

Khan (2014)

In the risk of bias summary (Figure 2, p. 14), Padhani marks "incomplete outcome data" for the Khan (2014) trial as "low risk".

In the characteristics of included studies, Padhani (p. 27) writes about the Khan (2014) trial:

“Incomplete outcome data (attrition bias): All outcomes: Low risk.
There was no loss to follow-up”

However, there is no flow diagram included in Khan (2014) and nor is there any statement in the text that there were no dropouts. Given this lack of information, there may or may not have been dropouts. It would be more appropriate to mark the assessment as “unclear risk”.

Yaqub (2015)

In the risk of bias summary (Figure 2, p. 14), Padhani marks “incomplete outcome data” for the Yaqub (2015) trial as “low risk”.

In the characteristics of included studies, Padhani (p. 31) writes about the Yaqub (2015) trial:
“Incomplete outcome data (attrition bias): Low risk.
No loss to follow-up”

Padhani (p. 15) also writes about the Yaqub (2015):
“We judged five studies to be at low risk for attrition bias as there were no reported losses to follow-up ... Yaqub 2015...”

Yaqub (2015, p. 210) wrote:
"If patient did not improve at all on first line antibiotic within 48 hours then antibiotic was changed to a 3rd generation cephalosporin i.e. Ceftriaxone (50 mg/kg/day) in 2 divided doses and patient was excluded from the study.”

Given this description by Yaqub, it seems highly likely that there were patients who were switched to the second antibiotic and therefore excluded. This type of exclusion can be associated with the effects of the intervention and is an unambiguous violation of the ITT principle. Padhani’s claim of ”no reported losses to follow-up” does not seem correct.

Pitt (1979) and Wahed (2008)

Padhani (p. 15) writes: *“We assessed two studies as at high risk of attrition bias: Pitt 1979 reported 21.6% loss to follow-up, and Wahed 2008 reported 30% loss to follow-up”*

However, Padhani does not consider the reasons for the drop outs.

Pitt (1979, p. 909) described:
“*Of the 862 recruits who began taking the pills, 64 recruits (34, vitamin C; 30, placebo) were removed from their platoons by the US Marine Corps for further training or for discharge during the eight-week study period. An additional 123 recruits (64, vitamin C; 59, placebo) were excluded from the final analysis because they did not continue to take their pills for the eight-week study period. One additional recruit was eliminated from the vitamin C group because of recurrent urticaria related to taking the tablets. The remaining 674 recruits (331, vitamin C; 343, placebo) completed the eight-week protocol and are the subject of analysis.*”

Thus, 64 drop outs were caused by administrative decisions at a higher level and the number of recruits removed is equal in both groups. It is unlikely that removal of these recruits was associated with the treatment. The 123 recruits who were excluded because they did not continue taking their pills is inconsistent with the ITT principle, but the number of excluded recruits is similar in both groups. For the vitamin C and placebo groups, the baseline variables are equal for age, race, previous medical history, geographic origin, and previous cold history (Table 1 in Pitt 1979). There is no evidence to suggest that these drop-outs substantially biased the comparison of the groups.

Wahed (2008, p. 78) wrote:
”*The children who left the hospital on ‘risk bond’ or ‘absconded’ from the ward or ‘expired’ during treatment were excluded from the analysis. Also those who developed a complication of pneumonia, were suffering from other severe systemic diseases with pneumonia, required antibiotics other than ampicillin and gentamysin and whose parents did not give consent for drawing blood were also excluded from the study.”*

These exclusions are quite different compared with the drop-outs in the Pitt (1979) trial, see above. If vitamin C had positive or negative effects in the Wahed (2008) trial, the effects could be substantially biased by the exclusions based on the disease progress.

Furthermore, in their Cochrane review, Hemilä (2013a, pp. 33-34) pointed out that, if the exclusions were random, it would be extremely unlikely to end up with exactly the reported 40 patients in 5 treatment groups, and 400 in the control group:
“*The authors state that “initially data was collected from 1150 children and after exclusions only 800 children were selected for analysis.” However, the original number of children in each of the 7 groups is not reported. When the reasons for exclusion seem to be random (complications of pneumonia etc.) it does not seem possible that random dropping out would lead to 5 groups which each had exactly 40 children and a [control] group which had exactly 400 children.”*

Thus, it is inappropriate to ignore the substantial differences in the reasons for the drop-outs in these two trials. It is much more likely that the drop-outs in the Wahed (2008) trial led to bias in the analysis, compared with the Pitt (1979) trial.

Selective reporting

Padhani writes: "*None of the studies provided any trial registration information or published protocols, therefore they were assessed as at unclear risk of reporting bias (Bancalari 1984; Coulehan 1974; Hunt 1994; Khan 2014; Pitt 1979; Wahed 2008; Yaqub 2015)*."

Publication of protocols is a recent phenomenon. Lack of a published protocol for earlier studies does not indicate that the selection of published outcomes was biased in the study reports. Selective reporting is a concern if there is a marginally significant difference in one published outcome between the treatment groups, and so there is a possibility that the particular outcome was selected from a group of outcomes on the basis of the lowest P-value. This concern is reasonable when a report actually publishes a marginally significant P-value. However, Padhani does not specifically describe any of the trials in which such a concern would arise.

Coulehan (1974) reported that there were no cases of pneumonia in the vitamin C or placebo groups - this cannot be selectively reported on the basis of the P-value.

Pitt (1979) reported cases of pneumonia in the vitamin C and placebo groups without publishing a P-value, so their decision to publish the number of cases cannot be based on finding a marginally significant difference.

Hunt (1994) published that "*with regard to clinical progress, 6 patients died during the trial - 5 placebo and 1 vitamin C. This difference was not significant statistically*" so that their decision to publish cannot be based on finding a marginally significant difference either.

Bancalari (1984) published the distribution of observed upper respiratory tract infections and Padhani does not explain whether the "unclear risk of selective reporting" means that Bancalari may have observed cases of pneumonia, but simply did not report any of them.

Overall assessment of the risk of bias in the included trials

There is no universal limit for the number of "high risk" items in the risk of bias assessment which should lead to a decision to exclude a trial. Nevertheless, in many cases a single "high risk" item may necessitate exclusion of the trial when there is concern that the risk of bias is considerable. However, in Padhani's risk of bias summary (Figure 2, p. 14), the Wahed (2008) trial has five items assessed as "high risk" and it seems inappropriate to include such a trial in the analysis. Nevertheless, the Wahed (2008) trial is given substantial importance even at the Abstract level, where Padhani summarizes the findings of the Wahed (2008) trial as follows: *"One study reported that the mean duration of hospital stay was 6.75 days amongst children in the vitamin C supplementation group and 7.75 days in the control group"*.

Fundamental misunderstanding about the assessment of potential bias

The risk of bias assessment is intended to guide an "author's judgment" of the potential risk of bias. The potential risk is not just dependent on what the original trial authors write in their Methods section, but the potential risk of bias also depends on what was found in the trial.

As an example, Padhani writes that in the Coulehan (1974) trial, there is "high risk" of bias in the item "random sequence generation (selection bias)". This is based on Padhani's quote in the included studies table (p. 23) *"All children were assigned alternately, from an alphabetical listing by classroom, to one of two study groups"*. However, Coulehan's statement should not be considered in isolation of the study findings.

Coulehan (1974, p. 7) reported that *"The school doctor or nurse treated 75 respiratory-illness episodes and 89 other illness episodes at the clinic during the 14-week period. None involved the lower respiratory tract"*. This lack of any lower respiratory tract infections indicates that there was no pneumonia. When there is not a single case of pneumonia in the trial, it is impossible to generate a biased estimate of the effect of vitamin C on pneumonia. Even if the allocation was extremely biased, such that all girls were in the vitamin C group and all boys in the placebo group for example, there would not be any bias in the recorded observation, which is 0 vs 0 cases of pneumonia. When there are no cases of pneumonia in the trial, even extremely unbalanced distributions of baseline variables cannot bias the comparison.

As a second example, Padhani writes that in the Hunt (1994) trial, there is "unclear risk" of bias in the item "Blinding of outcome assessment (detection bias)". Padhani (Analysis 2.1, p. 36) extracted Hunt's mortality data. There was one death in the vitamin C group, and five deaths in the placebo group. Poor blinding can cause bias in outcomes such as measuring pain or assessing X-ray images. However, death is an outcome that does not have any room for interpretation. It is not reasonable to consider that some deaths were misinterpreted because researchers knew to which group the dying patient belonged.

Errors and inconsistencies in data extraction

<u>Bancalari (1984) trial</u>

As described briefly in our main text, Padhani (2020) assumed that all 84 respiratory infections among 42 of the 62 participants reported by Bancalari (1984) were pneumonia, whereas not one of them was pneumonia. Figure 3 of Bancalari's report shows the distribution of the 84 respiratory infections and it is copied below.
Bancalari wrote:
"The most frequent type of ARI diagnosed in both grades was the common cold (Figure 3)"
["La IRA más frecuente diagnosticada en ambos cursos de escolares fue el Resfrío Común (Figura 3)"]

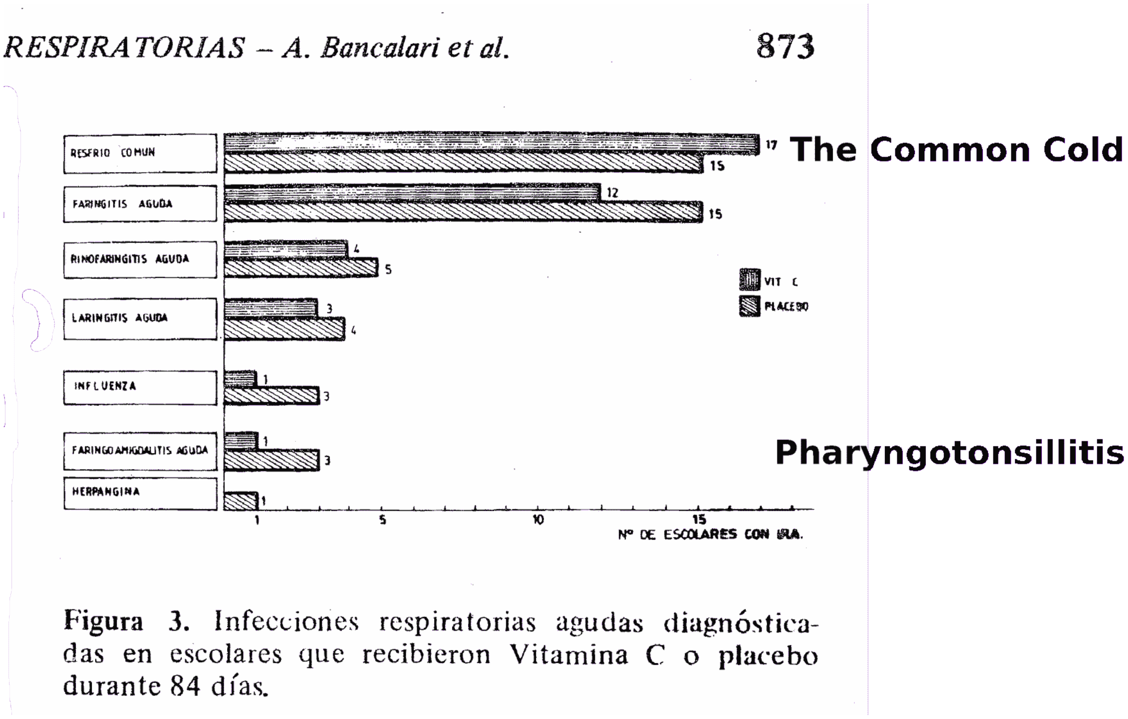

Bancalari (1984, p. 873):
Figura 3. Infecciones respiratorias agudas diagnosticadas en escolares que recibieron Vitamina C o placebo durante 84 dias.
[Figure 3. Acute respiratory infections diagnosed in schoolchildren who received vitamin C or placebos for 84 days]

Infections listed in Figure 3:

Resfrio Comun [the common cold] (Placebo group 15 cases / vitamin C 17 cases)
Faringitis aguda (Placebo group 15 cases / vitamin C 12 cases)
Rinofaringitis aguda (Placebo group 5 cases / vitamin C 4 cases)
Laringitis aguda (Placebo group 4 cases / vitamin C 3 cases)
Influenza (Placebo group 3 cases / vitamin C 1 case)
Faringoamigdalitis aguda [pharyngotonsillitis] (Placebo group 3 cases / vitamin C 1 case)
Herpangina (Placebo group 1 case / vitamin C 0 cases)

Placebo group total respiratory infections: 15 + 15 + 5 + 4 + 3 + 3 + 1
= 46 (= the published total cases in the placebo group)

Vitamin C group total respiratory infections: 17 + 12 + 4 + 3 + 1 + 1 + 0
= 38 (= the published total cases in the placebo group)

Not one of these 84 respiratory infections was pneumonia, though Padhani analyzes the data as if each child with a respiratory infection had pneumonia.

<u>Inconsistency in the extraction of outcomes that were not pre-specified</u>

There was inconsistency in the extraction of outcomes that were not pre-specified by Padhani.

In the Results section, Padhani extracted "number of days for improvement in oxygen saturation and respiratory rate" from the Khan (2014) trial. These are not outcomes pre-specified by Padhani and it is not evident that they should be presented with such prominence, if at all.

As the primary outcome in their trial, Hunt (1994) used 'total respiratory score', which had a range from three (least ill) to nine (most ill) and 10 (dead). This outcome is not mentioned by Padhani, though it is clinically relevant.

It is inconsistent to show the non-pre-specified outcomes by Khan (2014) but not to show the relevant but also non-pre-specified outcomes by Hunt (1994).

Errors and inappropriate methods in statistical calculations

Pooling trials which are clinically very different

"We conducted separate analyses to assess the impact of preventive and therapeutic supplementation of vitamin C and for child and adult populations" (Methods, p. 9).

"*We undertook meta-analyses only where this was meaningful, that is if the treatments, participants, and the underlying clinical question were sufficiently similar for pooling to generate meaningful conclusions*" (p. 11 left-hand column).

"*If the average treatment effect was not clinically meaningful, we did not combine the trials*" (p. 11 right-hand column).

However, this reasoning is not followed in Analysis 1.1 where Padhani pooled the Pitt (1979) and Bancalari (1984) trials. Analysis 1.1 is also shown as Figure 4, yet Figure 4 claims that it shows "outcome 2.1" (Mortality due to pneumonia" p. 36) whereas it actually shows "Outcome 1: Incidence of pneumonia" (from Analysis 1.1).

In Analysis 1.1 (Figure 4) Padhani pooled the Pitt (1979) trial in US Marine recruits (adults under extraordinary levels of physiological stress, most probably all males in the 1970s), with the Bancalari (1984) trial in 10-12 year old ordinary schoolchildren ("predominantly girls" according to the report). Bancalari et al. did not report any cases of pneumonia, but even if they had, the participants and conditions were so different that pooling of these two trials is not clinically meaningful.

Furthermore, in Padhani's Analysis 2.1 (Figure 4), the weight of the Bancalari (1984) trial with its 62 participants is 61.9%, whereas the weight of the Pitt (1979) trial with its 674 participants is just 38.1%. Consequently Pitt's results with actual cases of pneumonia are camouflaged by the large number of upper respiratory tract infections in the Bancalari trial.

It is very odd that Padhani claims in the Methods section (p. 9) that they *"conducted separate analyses … for child and adult populations"* given that they pooled the child and adult preventive trials (Figure 4 on p. 15; Analysis 1.1 on p. 36).

Erroneous calculation of the SD in the Bancalari (1984) trial

In the Abstract, Padhani describes the results of the Bancalari (1984) trial as follows: "*One study reported a decrease in the duration of illness in the vitamin C supplementation group (3.4 days ± 2.54) compared to the control group (4.5 days ± 2.35)."*

These same figures for the Bancalari (1984) trial are shown in the SOF table (p. 5), in the Results section (p. 16), and in Table 1 (p. 37).

Bancalari (1984) wrote "*During the 12 weeks of the study, there were 46 ARI episodes detected in the control group and 38 episodes in the vitamin C group (Figure 1). Moreover, in the group that received the vitamin C there was a significant decrease (p<0.05) in the duration of each ARI episode (3.4 days ± 0.45), compared with the control group (4.5 days ± 0.43) (Figure 2).*"

The dispersion estimates in Bancalari's text are SE. The SD can be calculated from them by multiplying with the square root of the number of observations. However, Padhani et al. (2020) used the wrong number of observations. They assumed that the unit of observation for the mean values was the person (N=32 in the vitamin C and N=30 in the placebo groups). Thereby Padhani calculated:

Vitamin C group: SD = sqrt(32)*0.45 = 2.546
Placebo group: SD = sqrt(30)*0.43 = 2.355

However, the text by Bancalari (1984) above clearly states that the unit of observation was "ARI episode" and therefore the correct SD values are:

Vitamin C group: SD = sqrt(38)*0.45 = 2.774
Placebo group: SD = sqrt(46)*0.43 = 2.916

The erroneous SD values are cited four times in the review. In addition, the recorded 38+46 ARI episodes were not pneumonia as previously described (pp. 23-24 of this Supplement).

Analysis of rare events

In Analysis 1.1 (Figure 4), Padhani shows the number of pneumonia cases recorded by Pitt (1979): 1/331 in the vitamin C group and 7/343 in the placebo group.
However, 1/331 is such a low incidence that the Mantel-Haenszel method used by Padhani is not appropriate.

Padhani writes:
"We used standard methodological procedures expected by Cochrane" (Abstract, p. 1).
"We rigorously followed Cochrane methods" (Discussion, p. 17).

However, the rare events section of the Cochrane Handbook states (2019, sect 10.4.4.3; yellow added):

"Simulation studies have revealed that many meta-analytical methods can give misleading results for rare events, which is unsurprising given their reliance on asymptotic statistical theory. Their performance has been judged suboptimal either through results being biased, confidence intervals being inappropriately wide, or statistical power being too low to detect substantial differences.
…
Bradburn and colleagues found that many of the most commonly used meta-analytical methods were biased when events were rare (Bradburn et al 2007). The bias was greatest in inverse variance and DerSimonian and Laird odds ratio and risk difference methods, and the Mantel-Haenszel odds ratio method using a 0.5 zero-cell correction. As already noted, risk difference meta-analytical methods tended to show conservative confidence interval coverage and low statistical power when risks of events were low.
At event rates below 1% the Peto one-step odds ratio method was found to be the least biased and most powerful method, and provided the best confidence interval coverage, provided there was no substantial imbalance between treatment and comparator group sizes within studies, and treatment effects were not exceptionally large. This finding was consistently observed across three different meta-analytical scenarios, and was also observed by Sweeting and colleagues (Sweeting et al 2004).
This finding was noted despite the method producing only an approximation to the odds ratio. For very large effects (e.g. risk ratio=0.2) when the approximation is known to be poor, treatment effects were

under-estimated, but the Peto method still had the best performance of all the methods considered for event risks of 1 in 1000, and the bias was never more than 6% of the comparator group risk."

Thus, the Peto method is recommended by the Cochrane Collaboration for the analysis of rare data such as the Pitt (1979) incidence with 0.3% (1/331) of participants having pneumonia in the vitamin C group.

The calculation method has a substantial effect on the confidence interval.
Using the inappropriate Mantel-Haenszel method, Padhani calculated that the effect of vitamin C in the Pitt (1979) trial to be RR=0.15 (95% CI: 0.02 to 1.20; Z = 1.79, P = 0.073, Analysis 1.1, p. 36).
In the earlier version of the Cochrane review on vitamin C and pneumonia, using the recommended Peto method, Hemilä (2013a) calculated that the effect of vitamin C in the Pitt (1979) trial was OR=0.23 (95% CI: 0.06 to 0.92; Z = 2.08, P = 0.038). This P-value by the Peto method is much closer to the mid-P = 0.044 (Hemilä 2013a; see also p. 28 of this Supplement).
The calculation of OR and RR using the Mantel-Haenszel method, and of OR using the Peto method are compared on the following page.

Padhani et al. also used the inappropriate Mantel-Haenszel method in their analysis of the occurrence of urticaria in the Pitt (1979) trial (1 vs 0 cases in Analysis 1.2, p. 36).
This led to RR = 3.11 (95% CI 0.13 to 75.03).
It is scientifically unsound to calculate and publish a RR based on a single case of urticaria. This estimate was even reported in the Abstract (p. 2): *"… the effect of vitamin C supplementation on ... adverse events (urticaria) (RR 3.11, 95% CI 0.13 to 76.03; 1 study, 674 participants… "*

The inappropriate Mantel-Haenszel method was also used in the analysis of mortality in the Hunt (1994) trial (1 vs 5 cases in Analysis 2.1, p. 36).

In the Methods section, Padhani (p. 11) writes:
"We entered outcome data for each study into data tables in Review Manager 5 to calculate treatment effects (Review Manager 2014). We used risk ratio (RR) for dichotomous outcomes"

There was no consideration given to the most appropriate method of analysis for rare events and no justification given for using the Mantel-Haenszel method.

In their previous Cochrane review on vitamin C and pneumonia, Hemilä (2013a, p. 6) considered in the Methods section:

"in the identified prophylactic trials the number of pneumonia cases in the vitamin C groups was very low (zero to two cases) and, therefore, we decided to use the Peto method for calculating the odds ratio (OR), which does not need corrections for zero cell counts (Higgins 2011).
Also, with only a few cases observed in the prophylactic trial groups, the mid-P value is the most appropriate method to calculate the P values for the differences in the treatment groups (Hemilä 2006; Lydersen 2009) and was used when comparing groups with small numbers of cases."

Calculation of the 95% CI and the P-value for the Pitt (1979) trial:

	Vitamin C		Placebo			Risk Ratio
Study or Subgroup	Events	Total	Events	Total	Weight	M-H, Fixed, 95% CI
Pitt (1979) Mantel-Haenszel RR	1	331	7	343	100.0%	0.15 [0.02, 1.20]
**Total (95% CI)**		**331**		**343**	**100.0%**	**0.15 [0.02, 1.20]**
Total events	1		7			
Heterogeneity: Not applicable						
Test for overall effect: Z = 1.79 (P = 0.07)						

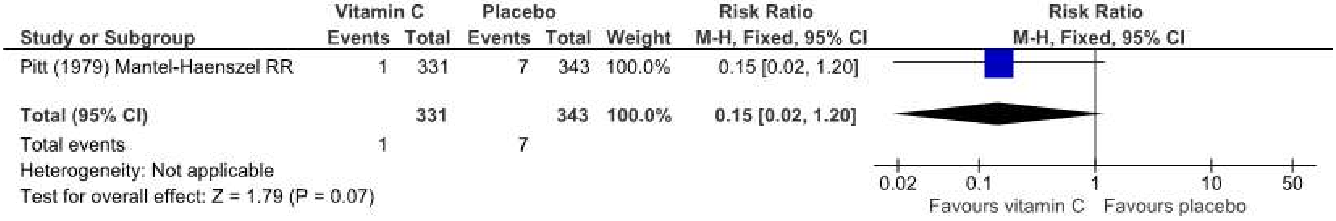

Z = 1.79 corresponds to P(2-tail) = 0.073

	Vitamin C		Placebo			Odds Ratio
Study or Subgroup	Events	Total	Events	Total	Weight	M-H, Fixed, 95% CI
Pitt (1979) Mantel-Haenszel OR	1	331	7	343	100.0%	0.15 [0.02, 1.19]
**Total (95% CI)**		**331**		**343**	**100.0%**	**0.15 [0.02, 1.19]**
Total events	1		7			
Heterogeneity: Not applicable						
Test for overall effect: Z = 1.80 (P = 0.07)						

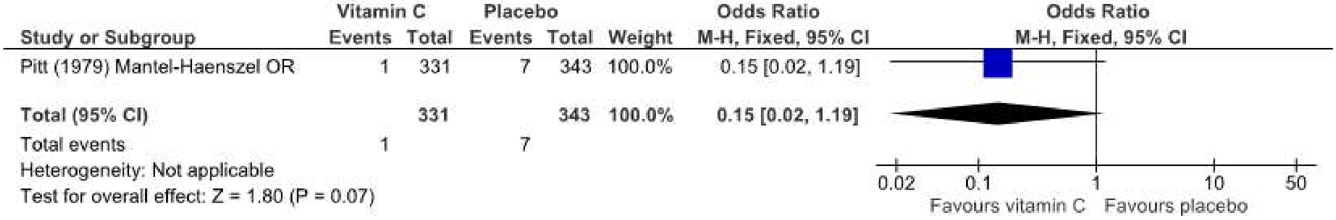

Z = 1.80 corresponds to P(2-tail) = 0.072

	Vitamin C		Placebo			Peto Odds Ratio
Study or Subgroup	Events	Total	Events	Total	Weight	Peto, Fixed, 95% CI
Pitt (1979) Peto method	1	331	7	343	100.0%	0.23 [0.06, 0.92]
**Total (95% CI)**		**331**		**343**	**100.0%**	**0.23 [0.06, 0.92]**
Total events	1		7			
Heterogeneity: Not applicable						
Test for overall effect: Z = 2.08 (P = 0.04)						

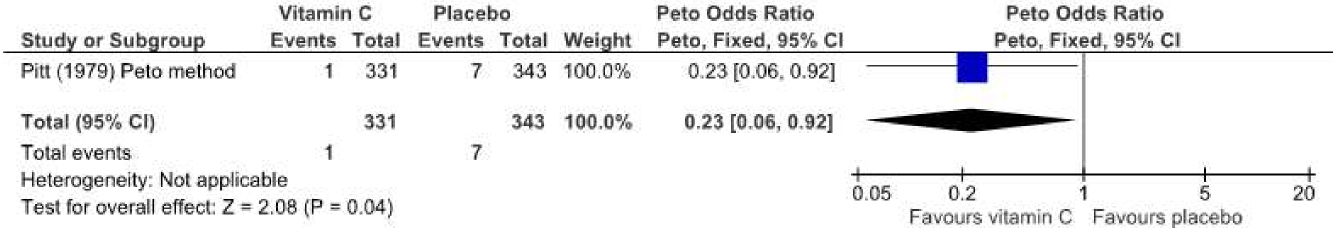

Z = 2.08 corresponds to P(2-tail) = 0.038

For very low counts of events, the exact methods are most appropriate for the calculation of the P-value and the 95% CI. Odds ratio estimation and confidence intervals using R:

```
> Pitt
     [,1] [,2]
[1,]    1  330
[2,]    7  336
> fisher.exact(Pitt, midp=T)
    Central Fisher's Exact Test (mid-p version)
data:  Pitt
p-value = 0.04
alternative hypothesis: true odds ratio is not equal to 1
95 percent confidence interval:  0.006 0.950
sample estimates: odds ratio    0.15
```

The upper 95% level (0.95) is much closer to the upper level of the Peto method (0.92), than to that of the Mantel-Haenszel RR method (1.20) (compare with the figures above). Consistently, the mid-P (0.04) is closer to the Peto-P (0.038) than to the Mantel-Haenszel-P (0.073).

Ignoring the inconsistency of the SD estimates in the Khan (2014) trial

Khan (2014, p. 56) published *"In Vitamin C group significant difference was observed between number of days consumed to show improvement in ... oxygen saturation (1.03± 0.16 mean ± SD) as compared to placebo group where days taken by … oxygen saturation to improve were (1.14± 1.0 mean ± SD)."*

The SD estimates 0.16 and 1.0 days are significantly different in the variance test.

The corresponding variances are 0.0256 and 1.0.
There were 111 participants in both groups, which gives 110 degrees of freedom.
These figures lead to F(110,110) = 39.1 (=1.0/0.0256), which corresponds to P = 10^{-57}. Thus, it is astronomically improbable that the SD estimates 0.16 and 1.0 from two series of 111 observations are compatible with random variation between samples of the same population.

Padhani did not pay attention to the inconsistency in the SD estimates in the Khan (2014) trial.
It is possible that there is an error in reporting (eg SD might be 1.16 or 1.6 instead of 0.16) or there could be errors in the calculations. However, this kind of inconsistency in the SD values should make the systematic reviewer cautious of other parts of the Khan (2014) report.

Further errors in the description of individual trials

The above text shows various problems in presenting and analyzing the included trials. This section highlights a few further concerns in Padhani's analysis that are not described above.

Coulehan (1974)

Padhani writes about the Coulehan (1974) trial:
"Doses of vitamin C supplementation used were ... 500 mg (Coulehan 1974)," (p. 12).
"The dose of supplementation was 500 mg in Coulehan 1974" (p. 15).
"For pneumonia prevention, the included studies provided supplementation in doses of 500 mg daily for 14 weeks, 2 g daily for 8 weeks, and 2 g daily for 12 weeks" (Abstract, p. 1).

In the Abstract, the 8-week and 12-week trials were Pitt (1979) and Bancalari (1984), respectively. The 14-week trial was Coulehan (1974).

However, Coulehan (1974, p. 7) wrote:
"The study period was 14 weeks from early February through mid-May. Children on vitamin C in Grades 1 through 4 (ages six to 10) received 1 g daily, whereas those in Grades 5 through 8 (ages 10 to 15) received 2 g."

Paradoxically, in the table describing characteristics of the included studies, Padhani gives the correct dosages of vitamin C for the Coulehan (1974) trial, however, most readers focus on the Abstract and Results sections, which are incorrect.

Bancalari (1984)

In the included studies table, Padhani (p. 21) writes about the Bancalari (1984) trial:
"Intervention (sample size): 2 g oral vitamin C in tablet form *twice weekly on Saturdays and Sundays for 12 weeks (N = 32)"*

However, Bancalari (1984) wrote:
"The vitamin C tablets (2 g per day) ... On Saturdays and Sundays, each child was supplied with the vitamin C or placebo tablets, following conversations with the parents or guardians, in order to ensure that the tablets were ingested properly."
[Original in Spanish: *"Vitamina C, que recibió 2 gr diarios (n = 32) ... Los fines de semana (sábado y domingo) se les entregó a cada niño la Vitamina C o el placebo previa conversación con los padres y/o apoderados, para asegurarnos de la ingestión"* (pp. 871-872).]

Thus, here Padhani has confused the arrangement for weekend vitamin C *distribution* with vitamin C *administration*.

Oddly enough, on page 15, Padhani correctly writes "The vitamin C tablets (2 g per day)"

Superficial description of the relevant literature

Relevant studies are ignored in background and discussion

The primary focus in a Cochrane review is the group of trials that are consistent with the inclusion criteria. It is these trials which are analyzed in the Results section. However, there is often highly relevant material independent of the included trials which should be described in the Background or Discussion sections, or both.

Cohort studies are usually excluded because residual bias is possible such that unknown confounders may explain differences between the control and exposure groups. However, if there is no association between the exposure and outcomes in a cohort study, it is unlikely that a genuine important effect is hidden by residual confounders. Therefore concern for bias is greater when strong associations are observed in a cohort study, and less or nil when there is no association between exposure and outcome.

In a cohort study Merchant (2004) found no association between vitamin C intake and community-acquired pneumonia in 38,378 middle-aged men in the USA. The number of pneumonia cases was 446 and therefore the null association has rather narrow 95% CI. This study seems highly relevant when considering the possible influence of vitamin C level in susceptibility to pneumonia in the general community. A cohort study of US women (Neuman 2007) also found no association between dietary vitamin C intake and the risk of pneumonia.. However, these studies were not mentioned in the Background or Discussion sections by Padhani. Differing study design is not the only potential explanation for the differences in findings between the Merchant (2004) study and the Pitt (1979) trial. Potential biological explanations for the divergence should also be considered (Hemilä 2013a).

The early reports indicating that vitamin C deficiency is frequently associated with pneumonia are also relevant background when considering the relationship between vitamin C and pneumonia (Hess 1920, Hemilä 2007). Although frank scurvy is currently rare, the increased occurrence of pneumonia in patients with scurvy indicates that in some contexts vitamin C may influence susceptibility to pneumonia. This is a relevant issue when considering biological plausibility, although observations of patients with severe vitamin C deficiency cannot be extrapolated to current Western populations.

Evidence of heterogeneity in antioxidant effects is ignored

As a background issue, when considering the effects of vitamin C on pneumonia, the evidence of heterogeneity in various antioxidant effects is important. In the ATBC Study, the effect of fat-soluble antioxidant vitamin E on the risk of pneumonia was significantly heterogeneous (Hemilä 2011a, 2016) and similar heterogeneity might occur for water-soluble vitamin C. In addition, there is strong evidence that the effect of vitamin C on the common cold (Hemilä 2013b), atrial fibrillation (Hemilä 2017), and on the duration of mechanical ventilation in the ICU (Hemilä 2020a) are significantly heterogeneous. Given these findings we should also expect heterogeneity in the effect of vitamin C on pneumonia. There is no justification to assume that a single RR estimate for vitamin C is applicable over the wider community. This background was not discussed by Padhani.

As described above, it seems plausible that the divergence between the Merchant (2004) study and the Pitt (1979) trial are explained by the differences in patients and their conditions, which would mean there is heterogeneity in the effect of vitamin C on pneumonia depending on the context.

Relevant data about safety is ignored

There are also highly relevant data about safety of vitamin C in studies that are not mentioned in Padhani's analysis. Two large-scale trials with 8,171 female health professionals and 14,641 male physicians found no adverse effects of 0.5 g/day of vitamin C when administered for 8 to 9 years, indicating long-term safety of such a dosage level (Cook 2007; Sesso 2008). In the US nutritional recommendations, the ‘tolerable upper intake level’ is stated to be 2 g/day of vitamin C for adults. However, the basis for this upper limit is the appearance of diarrhea in some individuals (IOM 2000) which is a trivial adverse effect that disappears quickly with a reduction in intake.

Padhani's conclusions on adverse effects of vitamin C are based on one single case of urticaria in the 2-month trial by Pitt (1979) with just 674 participants. Padhani does not discuss the safety reported in the above mentioned trials (which were much larger and longer), nor the conclusions of the IOM recommendations based on the thorough consideration of all the relevant literature on vitamin C. None of the trials included in Padhani's analysis used vitamin C doses higher than the ‘tolerable upper intake level’ of the US recommendations.

Lack of rigour in the Cochrane review on vitamin C for pneumonia

Among the Principles of the Cochrane Collaboration, is (Cochrane 2020a):

"Minimizing bias through a variety of approaches such as scientific rigour …"

The Cochrane Collaboration describes its vision as (Cochrane 2020b):

"Our vision is a world of improved health where decisions about health and health care are informed by high-quality, relevant and up-to-date synthesized research evidence."

Over one dozen authors and reviewers but none reading the text critically

This Cochrane review had 7 authors (Zahra Ali Padhani, Zorays Moazzam, Alina Ashraf, Hasana Bilal, Rehana A Salam, Jai K Das, Zulfiqar A Bhutta) and 5 named reviewers (Ann Fonfa, Theresa Wrangham, Bisi Oduwole, Robert Ware, and Mieke van Driel). In addition, one hopes that at least one of the editors of the Cochrane Acute Respiratory Infections Group would have read the review. Some of the errors that we describe in this commentary should be obvious to anyone who has a basic understanding of the epidemiology of respiratory infections.

For middle-aged Western adults, the incidence of pneumonia is about 1-3 cases per 1000 person-years (eg. Merchant 2004, Hemilä 2006). When Padhani shows in Figure 4 that 70% (21/30) of participants caught pneumonia during a 3 month period, alarm bells should ring for any critically minded reader and the origin of the Bancalari (1984) trial findings should have been checked. This incidence of pneumonia (70%) during a 12 week period corresponds to 2.6 cases of pneumonia per person per year. That is 2,600 cases per 1,000 person-years, which is three orders of magnitude higher than the incidence for Western adults. Although children in Chile possibly have a higher incidence of pneumonia compared to Western adults, it seems unlikely that the ratio is three orders of magnitude higher. It is concerning that none of the authors, reviewers, or editors read the text carefully enough to notice this problem. The Bancalari findings are not inconsequential in the review - they are reported in the only forest plot that is shown within the text section (Figure 4).

Although Cochrane review authors and reviewers need not all be professional statisticians, one expects that at least some people involved would be familiar with biostatistics at the level of the Cochrane Handbook (2019, sect 10.4.4.3): "*At event rates below 1% the Peto one-step odds ratio method was found to be the least biased and most powerful method, and provided the best confidence interval coverage, provided there was no substantial imbalance between treatment and comparator group sizes within studies, and treatment effects were not exceptionally large... For very large effects (e.g. risk ratio=0.2) when the approximation is known to be poor, treatment effects were under-estimated, but the Peto method still had the best performance of all the methods considered for event risks of 1 in 1000, and the bias was never more than 6% of the comparator group risk.*"

It is also concerning that apparently no-one wondered whether the analysis of comparisons of 1 vs 0 cases, 1 vs 5 cases, and 1 vs 7 cases, or of rate 1/331 was appropriate.

Although the majority of the problems we describe in this commentary require reading of the original reports, many of the problems such as those described above, should be apparent without reading the original reports. However, some of the review authors should also have read the original reports and therefore the large number of concerns that we describe here is worrying.

Furthermore, the Methods section of the Padhani review states: "*Four review authors (ZAP, ZM, AA, HB) extracted the data*" and "*Four review authors (ZAP, ZM, AA, HB) independently assessed the risk of bias for each study … Any disagreements were resolved by discussion, and tables cross-checked by another review author (RAS)*."

Previous problems in the lack of rigour in Cochrane reviews

The Cochrane review on vitamin C and pneumonia is not unique in its lack of rigour.

Hemilä (2011b) listed 10 substantial problems in the Cochrane review "zinc for the common cold" by Singh (2011). However, most of these problems were still present in the updated review by Singh (2013) and serious new errors were introduced (Hemilä 2015). The review was eventually retracted because of plagiarism (Singh 2015). Thus, the editorial processes in that review were also far from rigorous as the problems described in the 2011 version were not considered by the Cochrane editors before publishing the update as Singh (2013).

A third case in which rigour of the editorial processes was lacking was the Cochrane review on "vitamin C and asthma" by Kaur (2009). Hemilä (2009,2013c) pointed out that there were obvious errors in the extraction of data and in the calculations. As a response to that critique, the Cochrane editors removed three Analysis figures of that review. Analysis figures are the scientific core of the Cochrane reviews and removal of them is unambiguously against the COPE guidelines (COPE 2020a, 2020b). Although the editor in chief, David Tovey, was informed of the removal of the three Analysis figures, he was unwilling to make the original version of the Cochrane review publicly available. Finally, Hemilä contacted COPE and described the removal of the figures and COPE stated that the original version needed to be made available. As a result of that process, there are now unfortunately three different versions of that Cochrane review with one single PubMed identification code (Hemilä 2020b).

Thus, reviews published by the Cochrane Collaboration are not consistently scientifically rigorous.

References

Bancalari (1984)
Bancalari A, Seguel C, Neira F, Ruíz I, Calvo C.
Prophylactic value of vitamin C in acute respiratory tract infections in schoolchildren.
Revista Médica de Chile 1984;112:871-6.
https://pubmed.ncbi.nlm.nih.gov/6398492
https://www.mv.helsinki.fi/home/hemila/CC/Bancalari_1984_ch.pdf
https://www.mv.helsinki.fi/home/hemila/CC/Bancalari_1984_bm.pdf
https://www.mv.helsinki.fi/home/hemila/T6.pdf [Translation to English]

Cochrane (2020a)
Principles of Collaboration: Working Together for Cochrane.
https://community.cochrane.org/organizational-info/resources/policies/policies-all-members-and-supporters/principles-collaboration-working-together-cochrane (Accessed July 15, 2020).

Cochrane (2020b)
About us: Our vision.
https://www.cochrane.org/about-us (Accessed July 15, 2020).

Cochrane Handbook (2019)
Higgins J, Thomas J (ed). Cochrane Handbook for Systematic Reviews of Interventions.
https://training.cochrane.org/handbook/current

Cook (2007)
Cook NR, Albert CM, Gaziano JM, Zaharris E, MacFadyen J, Danielson E, et al.
A randomized factorial trial of vitamins C and E and beta carotene in the secondary prevention of cardiovascular events in women: results from the Women's Antioxidant Cardiovascular Study.
Archives of Internal Medicine 2007;167:1610–8.
https://doi.org/10.1001/archinte.167.15.1610

COPE (2020a)
Committee of Publication Ethics (COPE).
Retraction Guidelines.
https://publicationethics.org/files/retraction-guidelines.pdf (Accessed March 30, 2020).

COPE (2020b)
Committee of Publication Ethics (COPE).
A Short Guide to Ethical Editing for New Editors.
https://publicationethics.org/resources/guidelines-new/short-guide-ethical-editing-new-editors (Accessed March 30, 2020).

Coulehan (1974)
Coulehan JL, Reisinger KS, Rogers KD, Bradley DW.
Vitamin C prophylaxis in a boarding school.
New England Journal of Medicine 1974; 290:6-10.
https://doi.org/10.1056/NEJM197401032900102
https://www.ncbi.nlm.nih.gov/pubmed/787788

Glazebrook (1942)
Glazebrook AJ, Thomson S.
The administration of vitamin C in a large institution and its effect on general health and resistance to infection.
Journal of Hygiene 1942;42:1-19.
https://www.ncbi.nlm.nih.gov/pmc/articles/PMC2199803

Gorton (1999)
Gorton H, Jarvis K.
The effectiveness of vitamin C in preventing and relieving the symptoms of virus-induced respiratory infections.
Journal of Manipulative and Physiological Therapeutics 1999;22:530–3.
https://doi.org/10.1016/s0161-4754(99)70005-9
https://www.ncbi.nlm.nih.gov/pubmed/10543583

Hemilä (1997)
Hemilä H.
Vitamin C intake and susceptibility to pneumonia.
Pediatric Infectious Disease Journal 1997;16:836–7.
https://doi.org/10.1097/00006454-199709000-00003
https://helda.helsinki.fi/handle/10138/225875

Hemilä H (2004)
Hemilä H, Virtamo J, Albanes D, Kaprio J.
Vitamin E and beta-carotene supplementation and hospital-treated pneumonia incidence in male smokers.
Chest 2004;125:557-65.
https://doi.org/10.1378/chest.125.2.557

Hemilä (2007)
Hemilä H, Louhiala P. Vitamin C may affect lung infections.
Journal of the Royal Society of Medicine 2007;100:495-8.
https://doi.org/10.1258/jrsm.100.11.495
https://www.ncbi.nlm.nih.gov/pmc/articles/PMC2099400

Hemilä (2009)
Hemila H.
Feedback to Cochrane review on vitamin C and asthma
[Comments on the Cochrane review “vitamin C supplementation for asthma” by Kaur B, Rowe BH, Stovold E]
Repository of the University of Helsinki
https://helda.helsinki.fi/handle/10138/38500

Hemilä (2011a)
Hemilä H, Kaprio J.
Subgroup analysis of large trials can guide further research: a case study of vitamin E and pneumonia.
Clinical Epidemiology 2011;3:51–9.
https://doi.org/10.2147/CLEP.S16114
https://www.ncbi.nlm.nih.gov/pmc/articles/PMC3046185

Hemilä (2011b)
Hemilä H.
The zinc for the common cold review by Singh and Das has a number of problems which should be considered when the review is next time updated [Feedback on Cochrane review "Zinc for the common cold"].
Repository of the University of Helsinki
https://helda.helsinki.fi/handle/10138/39188

Hemilä (2013a)
Hemilä H, Louhiala P.
Vitamin C for preventing and treating pneumonia.
Cochrane Database of Systematic Reviews 2013;(8):CD005532
https://doi.org/10.1002/14651858.CD005532.pub3
https://helda.helsinki.fi/handle/10138/225862
https://www.mv.helsinki.fi/home/hemila/CP/2013_Coch_Pneu_CD005532.pdf

Hemilä (2013b)
Hemilä H, Chalker E.
Vitamin C for preventing and treating the common cold.
Cochrane Database of Systematic Reviews 2013;(1):CD000980
https://doi.org/10.1002/14651858.CD000980.pub4
https://helda.helsinki.fi/handle/10138/225864
https://www.mv.helsinki.fi/home/hemila/CC/2013_Coch_Colds_CD000980.pdf

Hemilä (2013c)
Hemila H.
Vitamin C and exercise-induced bronchoconstriction: further problems in the Cochrane review "vitamin C for asthma" (2013).
Repository of the University of Helsinki
https://helda.helsinki.fi/handle/10138/40816

Hemilä (2015)
Hemilä H.
Concerns about unattributed copying of text and data, and about numerous other problems in the Cochrane review "Zinc for the Common Cold" by Singh M, Das RR (2013).
Repository of the University of Helsinki
https://helda.helsinki.fi/handle/10138/153180

Hemilä (2016)
Hemilä H.
Vitamin E and the risk of pneumonia: using the I^2-statistic to quantify heterogeneity within a controlled trial.
British Journal of Nutrition 2016;116:1530–6.
https://doi.org/10.1017/S0007114516003408

Hemilä (2017)
Hemilä H, Suonsyrjä T.
Vitamin C for preventing atrial fibrillation in high risk patients: a systematic review and meta-analysis.
BMC Cardiovascular Disorders 2017;17:49
https://doi.org/10.1186/s12872-017-0478-5
https://www.ncbi.nlm.nih.gov/pmc/articles/PMC5286679

Hemilä (2020a)
Hemilä H, Chalker E.
Vitamin C may reduce the duration of mechanical ventilation in critically ill patients: a meta-regression analysis.
Journal of Intensive Care 2020;8:15
https://doi.org/10.1186/s40560-020-0432-y
https://www.ncbi.nlm.nih.gov/pmc/articles/PMC7006137

Hemilä (2020b)
Hemilä H.
Cochrane has not consistently followed the COPE guidelines.
European Journal of Clinical Investigation 2020;50:e13216.
https://doi.org/10.1111/eci.13216

Hess (1920)
Hess AF. Scurvy: Past and Present. Philadelphia, PA: Lippincott, 1920:88, 99.
http://chla.library.cornell.edu
https://archive.org/details/b29823778/page/n4/mode/2up

Hunt (1994)
Hunt C, Chakravorty NK, Annan G, Habibzadeh N, Schorah CJ.
The clinical effects of vitamin C supplementation in elderly hospitalised patients with acute respiratory infections.
International Journal for Vitamin and Nutrition Research 1994;64:212-9.
https://www.ncbi.nlm.nih.gov/pubmed/7814237
https://www.mv.helsinki.fi/home/hemila/CP/Hunt_1994_ch.pdf
https://www.mv.helsinki.fi/home/hemila/CP/Hunt_1994_bm.pdf

IOM (2000)
Institute of Medicine.
Vitamin C. Dietary Reference Intakes for Vitamin C, Vitamin E, Selenium and Carotenoids.
Washington DC: National Academy Press, 2000:95–185.
https://pubmed.ncbi.nlm.nih.gov/25077263
https://www.nap.edu/catalog/9810/dietary-reference-intakes-for-vitamin-c-vitamin-e-selenium-and-carotenoids

Kaur (2009)
Kaur B, Rowe BH, Arnold E.
Vitamin C supplementation for asthma.
Cochrane Database of Systematic Reviews 2009;(1):CD000993
Three different versions (see explanation in Hemilä 2020b):

1. Original with the three Analysis figures present, but Hemilä (2009) feedback missing
https://www.ncbi.nlm.nih.gov/pmc/articles/PMC6176494

2. Version with the three Analysis figures present and Hemilä (2009) feedback on p. 27
https://helda.helsinki.fi/handle/10138/296440

3. Version with the three Analysis figures removed, but Hemilä (2009) feedback remaining
https://doi.org/10.1002/14651858.cd000993.pub3

The PubMed record links to the first and the third versions described above:
https://pubmed.ncbi.nlm.nih.gov/19160185

Khan (2014)
Khan IM, Shabbier A, Naeemullah S, Siddiqui FR, Rabia M, Khan SN, Chaudhary MT
Efficacy of vitamin C in reducing duration of severe pneumonia in children.
Journal of Rawalpindi Medical College 2014;18:55-7.
https://www.journalrmc.com/index.php/JRMC/article/view/381
https://www.journalrmc.com/volumes/1405749894.pdf

Mahalanabis (2006b)
Mahalanabis D, Jana S, Shaikh S, Gupta S, Chakrabarti ML, Moitra P, et al.
Vitamin E and vitamin C supplementation does not improve the clinical course of measles with pneumonia in children: a controlled trial.
Journal of Tropical Pediatrics 2006; 52:302-3.
https://doi.org/10.1093/tropej/fmi100

Merchant (2002)
Merchant AT, Curhan G, Bendich A, Singh VN, Willett WC, Fawzi WW.
Vitamin intake is not associated with community-acquired pneumonia in U.S. men.
Journal of Nutrition 2004;134:439-44.
https://doi.org/10.1093/jn/134.2.439

Neuman (2007)
Neuman MI, Willett WC, Curhan GC.
Vitamin and micronutrient intake and the risk of community-acquired pneumonia in US women.
American Journal of Medicine 2007;120:330-6.
https://doi.org/10.1016/j.amjmed.2006.06.045

Padhani (2020)
Padhani ZA, Moazzam Z, Ashraf A, Bilal H, Salam RA, Das JK, Bhutta ZA.
Vitamin C supplementation for prevention and treatment of pneumonia.
Cochrane Database of Systematic Reviews 2020;(4):CD013134.
https://doi.org/10.1002/14651858.CD013134.pub2

Pitt (1979)
Pitt HA, Costrini AM.
Vitamin C prophylaxis in marine recruits.
JAMA 1979;241:908-11.
https://doi.org/10.1001/jama.1979.03290350028016

Sesso (2008)
Sesso HD, Buring JE, Christen WG, Kurth T, Belanger C, MacFadyen J, et al.
Vitamins E and C in the prevention of cardiovascular disease in men: the Physicians’ Health Study II randomized controlled trial.
JAMA 2008;300: 2123–33.
https://doi.org/10.1001/jama.2008.600

Singh (2011)
Singh M, Das RR.
Zinc for the common cold.
Cochrane Database of Systematic Reviews 2011;(2):CD001364.
https://doi.org/10.1002/14651858.cd001364.pub3

Singh (2013)
Singh M, Das RR.
Zinc for the common cold.
Cochrane Database of Systematic Reviews 2013;(6):CD001364.
https://doi.org/10.1002/14651858.cd001364.pub4

Singh (2015)
Singh M, Das RR.
WITHDRAWN: Zinc for the common cold.
Cochrane Database of Systematic Reviews 2015;(4):CD001364.
https://doi.org/10.1002/14651858.cd001364.pub5

Wahed (2008)
Wahed MA, Islam MA, Khondakar P, Haque MA.
Effect of micronutrients on morbidity and duration of hospital stay in childhood pneumonia.
Mymensingh Medical Journal 2008;17:S77-83.
https://www.ncbi.nlm.nih.gov/pubmed/18946457

Yaqub (2015)
Yaqub A, Riaz N, Ghani Z, Gul S.
Role of vitamin C in children having pneumonia.
ISRA Medical Journal 2015;7:209-11.
https://www.pakmedinet.com/25512
http://www.imj.com.pk/role-of-vitamin-c-in-children-having-pneumonia
http://www.imj.com.pk/wp-content/uploads/2015/12/ROLE-OF-VITAMIN-C-IN-CHILDREN-HAVING-PNEUMONIA.pdf
